# Data on ion composition and X-ray diffraction patterns of biosolids from wastewater treatment plants in Lufkin and Nacogdoches, Texas, USA

**DOI:** 10.1016/j.dib.2018.08.087

**Published:** 2018-08-30

**Authors:** Kefa K. Onchoke, Christopher M. Franclemont, Paul W. Weatherford

**Affiliations:** a Department of Chemistry & Biochemistry, Stephen F. Austin State University, Box 13006 – SFA Station, Nacogdoches, TX, 75962-13006, USA; b Department of Agriculture, Stephen F. Austin State University, Soil, Plant and Water Analysis Lab, P.O. Box 13025 SFA Station, Nacogdoches, TX 75962-3025, USA

## Abstract

The data presented in this article is related to the research article entitled, “Structural Characterization and Evaluation of Municipal Wastewater Sludge (Biosolids) from two Rural Wastewater Treatment Plants in East Texas, USA” (Onchoke et al., [1]). The XRD profiles and composition of biosolids from two wastewater treatment plant is presented. This study describes the composition of XRD crystalline phase patterns of the wastewater sludge. After the removal of the Kα_2_ peaks the d-spacing and hkl values were determined. In addition, the ion chromatographic profile of the seven anions ($N{O_{3}}^{-}$, $N{O_{2}}^{-}$, Br^−^, Cl^−^, F^−^, $S{O_{4}}^{2-}$, and $P{O_{4}}^{3-}$) in biosolids is presented.

**Specifications Table**

**Table:** 

Subject area	*Environmental Chemistry*
More specific subject area	*Wastewater sludge (biosolids)*
Type of data	*Table, graph, figure*
How data was acquired	*Ion chromatography, XRD, SEM, EDX were used in the study.*
	(a) Dionex Integrion HPIC ion chromatograph (Thermo Fisher Scientific Inc., USA) was used for anion analysis. (b) A Bruker AXS D8 Advance diffractometer equipped with an X-ray tube (Cu K_α_ radiation: λ = 1.54060 Å, 40 kV, and 40 mA) using a Ni filter and one-dimensional LynxEye detector at scanning speed of 2 °/min and 0.0125 ° step sizes and a 1 s/step. (c) A JEOL-JSM 6100 scanning electron microscope equipped with a Horiba energy dispersive X-ray spectroscopy (SEM/EDX) was used.
Data format	Raw, filtered, analyzed
Experimental factors	(a) For XRD analysis: Biosolid samples were obtained from Nacogdoches and Lufkin wastewater treatment plant (NWWTP, LWWTP), air dried, and ground to powder. (b) For IC analysis: samples were filtered on a 0.45 μm filter.
Experimental features	Wastewater sludge generated from the rural municipal wastewater treatment plants are applied for land. We provide the characterization of the crystalline phases in the biosolids. The powder diffraction file was acquired using Bruker AXS DIFFRAC.EVA program [2]. The fitted line profiles, peak search methods, and indexing of the lines were used to calculate the mineral identification via comparisons with the diffraction patterns with TOPAS program [3].
Data source location	Nacogdoches, East Texas, in East Texas, USA latitude: 31° 33′ 31.2444′′ N and longitude 94° 38′ 52.1808′′ W,
Data accessibility	All data are available within this article.
Related research article	Associated Paper: “Structural Characterization and Evaluation of Municipal Wastewater Sludge (Biosolids) from two Rural Wastewater Treatment Plants in East Texas, USA”, Onchoke, K.K, Franclemont, C.M., Spectrochim Acta A, In press [1]

**Value of the data**

• The data provided here is important for wastewater and wastewater treatment plants, water resources. The data provides important information for identification of elemental compositions in biosolids.
• The indexed hkl and d-spacing values can be used for referencing and identification of crystalline phases prevalent in biosolids/wastewater sludge.
• The XRD patterns are important for the identification of any newer crystalline phases in wastewater treatment plants, and in particular in East Texas. This data can also be used for comparisons to other wastewater treatment plants. The data serves as a benchmark for other researchers analyzing biosolids generated from wastewater treatment plants.

## 1 Data

Wastewater treatment plants generate large amounts of wastewater sludge (also known as biosolids) [4]. Wastewater biosolids can be disposed of in several ways, namely, for enrichment of soils, or for landfills [5], [6], [7], [8]. The data in this paper presents information on the crystalline phases, their approximate compositions, their d-spacings and hkl patterns (Fig. 3A and B, and Table 1, Table 2). An ion chromatographic profile with parameters used for analysis of seven anions (Cl^−^, F^−^, $NO_{3}^{-}$ , $NO_{2}^{-}$, Br^-^, $SO_{4}^{2-}$, and $PO_{4}^{3-}$ ) during the analyses is provided (Fig. 2).

**Table 1:** Analysis of the Crystalline Phases, d-spacing, and h, k, l values of biosolids in NWWTP [1].

Index	Angle *(2θ)*	*d*-Value	Net Intensity	Gross Intensity	Rel. Intensity	*h*, *k*, *l*	Mineral
0	6.217	14.20528	2756	10,258	31.40%	0 0 2	Vermiculite
1	20.756	4.27606	1703	6741	19.40%	1−2 −1	Alunogen
						1 −2 −1	Hexahydrite
						1−2−1	Quartz
2	22.663	3.92039	258	4887	2.90%	0 3 1	Gypsum
3	25.201	3.53096	255	4588	2.90%	2 2 1	Laumontite
4	26.422	3.37054	3254	7472	37.10%	3 1 2	Laumontite
5	26.53	3.35703	8779	12,982	100.00%	0 1 1	Quartz
6	27.266	3.26809	341	4425	3.90%	1 0 1	Quartz
7	27.359	3.25717	296	4365	3.40%	1 1 6	Vermiculite
						1 -3 1	Mirabilite
8	27.886	3.19679	225	4182	2.60%	1 1 2	Annite Mica
9	31.569	2.83182	196	3637	2.20%	0 2 8	Vermiculite
10	32.373	2.7633	212	3602	2.40%	2 −3 −1	Gypsum
11	35.837	2.50374	274	3498	3.10%	3 2 −3	Vermiculite
						1 3 3	Antigorite T
						1 −3 1	Talc
12	35.963	2.49521	171	3401	1.90%	1 3 −11	Antigorite T
13	36.148	2.48288	157	3389	1.80%	1 -3 2	Annite Mica
14	36.39	2.46693	501	3726	5.70%	1 1 0	Quartz
15	38.122	2.3587	157	3189	1.80%	0 0 3	Kaolinite
16	38.325	2.34672	160	3168	1.80%	1 -3 1	Kaolinite
17	39.336	2.28866	502	3449	5.70%	1 0 2	Quartz
						0 1 2	
18	40.173	2.24291	243	3181	2.80%	1 1 1	Quartz
19	42.263	2.13671	265	3079	3.00%	0 2 0	Quartz
20	45.719	1.98288	275	2919	3.10%	2 0 1	Quartz
						0 2 1	
21	48.994	1.85774	131	2513	1.50%	0 6 2	Laumontite
22	49.996	1.82283	1566	3964	17.80%	1 1 2	Quartz
23	55.225	1.66197	145	2426	1.70%	0 1 3	Quartz
24	59.809	1.54506	598	2789	6.80%	2 1 1	Quartz
						1 2 1	
25	67.57	1.38524	305	2359	3.50%	1 2 2	Quartz
						2 1 2	
26	68.019	1.37718	781	2833	8.90%	0 2 3	Quartz
						2 0 3	
27	68.16	1.37468	553	2596	6.30%	3 0 1	Quartz
						0 3 1	
28	73.358	1.28957	239	2203	2.70%	0 1 4	Quartz
						1 0 4	
29	75.597	1.25684	393	2319	4.50%	0 3 2	Quartz
						3 0 2	
30	79.662	1.20261	271	1528	3.10%	2 1 3	Quartz
						1 2 3

**Table 2:** Analysis of the crystalline phases, d-spacing, and h, k, l values of biosolids in LWWTP [1].

Index	Angle *(2θ)*	*d* Value	Net Intensity	Gross Intensity	Rel. Intensity	*h k l*	Mineral
0	6.196	14.25293	2843	10,629	36.20%	0 0 2	Vermiculite
1	11.645	7.59308	554	4410	7.10%	0 2 0	Gypsum
2	19.881	4.46235	524	4195	6.70%	1 0 1	Andalusite
3	20.825	4.26215	1469	5156	18.70%	0 1 0	Quartz Gypsum
						1 −2 −1	
4	25.287	3.51915	198	3358	2.50%	2 2 1	Laumontite
5	26.622	3.34568	7847	10,898	100.00%	0 1 1	Quartz
						1 0 1	
6	27.008	3.29879	313	3309	4.00%	2 4 1	Palygorskite
7	27.737	3.21372	792	3667	10.10%	2 2 1	Palygorskite
8	29.104	3.06578	478	3225	6.10%	1 4 −1	Gypsum
9	31.083	2.87493	297	2862	3.80%	2 -2 -2	Vermiculite,
						3 −1 −4	Hexahydrite
10	31.761	2.81512	131	2644	1.70%	1 1 1	Laumonthite
						3 −1 −4	
11	33.327	2.68633	225	2515	2.90%	1 −3 −1	Annite Mica
12	33.411	2.67978	139	2421	1.80%	0 6 1	Vermiculite
13	35.938	2.49691	150	2436	1.90%	2 0 -2	Actolite
14	36.5	2.45972	537	2768	6.80%	1 3 4	Vermiculite
						1 1 0	Quartz
15	37.734	2.3821	150	2246	1.90%	1 1 0	Corundum
16	39.426	2.28366	479	2517	6.10%		Quartz Vermiculite
17	40.251	2.23874	216	2246	2.80%	1 1 1	Quartz + Palygorskite
						4 −2 −2	
18	42.44	2.12817	254	2218	3.20%	3 5 1	Quartz + Palygorskite
19	43.236	2.09083	121	2029	1.50%	0 2 0	Quartz
20	43.647	2.07209	141	2014	1.80%	1 1 3	Corundum
21	45.571	1.989	126	1929	1.60%	2 0 1	Hexahydrite
						0 2 1	
22	45.769	1.98085	274	2070	3.50%	2 0 1	Quartz
						0 2 1	
23	46.944	1.93396	125	1844	1.60%	3 −1 1	Turquoise
24	47.998	1.89391	137	1818	1.70%	0 0 2	Boehemite
25	48.374	1.88009	106	1778	1.30%	4 0 −4	Laumonite
26	50.115	1.81878	758	2421	9.70%	1 1 2	Quartz
27	54.851	1.6724	318	1872	4.10%	2 0 2	Quartz
						0 2 2	
28	59.932	1.54218	513	1930	6.50%	1 2 1	Quartz
						2 1 1	
29	67.736	1.38224	357	1704	4.50%	1 2 2	Quartz
						2 1 2	
						4 4 0	
30	68.112	1.37553	603	1954	7.70%		Quartz Vermiculite
31	68.106	1.37564	625	1976	8.00%		Quartz Vermiculite
32	73.466	1.28794	234	1546	3.00%	014	Quartz
						104	
33	75.625	1.25645	133	1320	1.70%	032	Quartz
						302	
34	75.702	1.25536	88.4	1266	1.10%	032	Quartz
35	85.016	1.14001	20.7	66.3	0.30%	204	Quartz
						024	

## 2 Experimental design, materials, and methods

The Experimental methods and procedures that allowed the data here presented are described in Ref. [1] and in cited references. Here, only the protocol for XRD and SEM morphological analysis is provided, giving a large number of experimental details, usually omitted in research articles due to the words limit.

### 2.1 Study area description

**Fig. 1:**
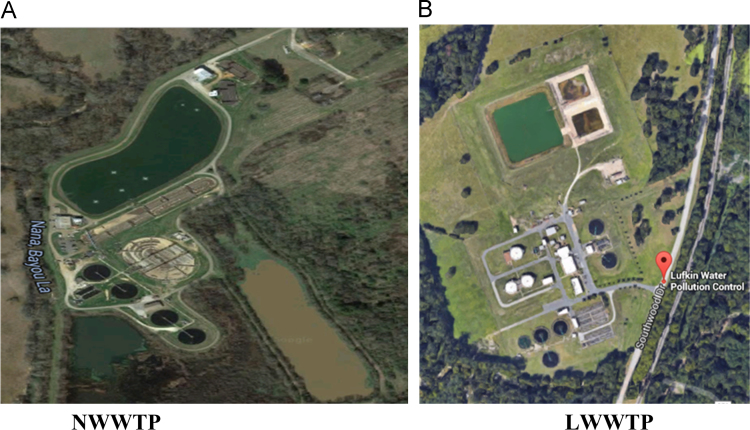
Aerial photographs of (a) Nacogdoches wastewater treatment plant (NWWTP), and (b) Lufkin wastewater treatment plant (LWWTP). In each of the pictures, 4 clarifiers are observed.

The Nacogdoches and Lufkin Wastewater Treatment Plants (NWWTP, LWWTP, shown in Fig. 1) are located in Nacogdoches City (Population: 33, 000) and Lufkin City (Population: ~35,000). These wastewater treatment plants are activated wastewater treatment plants. The NWWTP and LWWTP have wastewater treatment capacity of 12.88 million gallons per day (MGD) 11.3 MGD, respectively.

### 2.2 Sampling and collection of biosolids

Biosolids were collected from the Nacogdoches Wastewater Treatment Plant (NWWTP) and Lufkin Wastewater treatment Plant ( LWWTP) during the Summer 2016 and 2017. Multiple grab samples from the Nacogdoches and Lufkin WWTPs were dried in the lab. Biosolid samples were stored in plastic containers or 5-gallon plastic buckets. Proper care was taken to avoid any contamination during each sampling period.

### 2.3 Ionic analysis elution profile

Approximately 28 mg of finely crushed biosolids was first added to a 25 mL volumetric flask and the volume filled to the mark using 18.2 MΩ water. The contents of the flasks were then sonicated for 20 min and the sample split into two separate 15 mL Falcon tubes. Following this, the samples were centrifuged at 7650 rpm for 20 minutes and filtered through 0.45 μm filters. Samples were then analyzed with anion chromatography.

### 2.4 Morphological characterization of biosolids

The biosolids were air dried, crushed with mortar and pestle, and analyzed with JEOL-JSM 6100 scanning electron microscope equipped with a Horiba energy dispersive X-ray spectroscopy (SEM/EDX) with an accelerating voltage of 15 kV. The surface morphology, particle diameters (Fig. 4, Fig. 5, Fig. 6) of biosolids were measured at X40, 100 - 200× magnifications. Powder XRD analysis was performed in the 2*θ* range of 2° –90° on a Bruker AXS D8 Advance diffractometer equipped with an X-ray tube (Cu K_α_ radiation: *λ* = 1.54060 Å, 40 kV, and 40 mA) using a Ni filter and one-dimensional LynxEye detector at scanning speed of 2 °/min and 0.0125 ° step sizes and a 1 s/step. The diameters of select pores (Fig. 6) were measured at 1–5 k magnification. Powder XRD patterns (Fig. 3, Fig. 4, Fig. 5) and their hkl values was used to identify the crystalline structural phases present in biosolids (Tables 1 and 2).

**Fig. 2:**
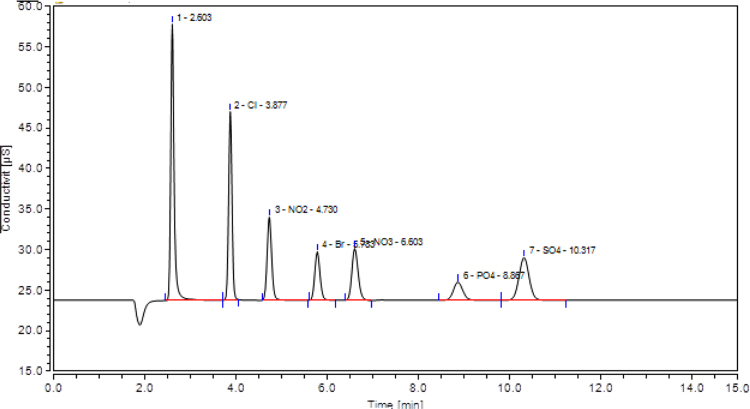
Representative elution profile of the seven anions and retention times. 1 = F^−^, 2 = Cl^−^, 3 = NO_2_^−^, 4 = Br^−^, 5 = NO_3_^−^, 6 = PO_4_^3−^, 7 = SO_4_^2−^. The standard was diluted X20 times. The Dionex Integrion HPIC ion chromatograph (Thermo Fisher Scientific Inc., USA) was used. A Dionex IonPac AS22 analytical column (2 × 250 mm) thermostated at 30 °C, guard column (IonPac AG22), a Dionex AS 22 Eluent Concentrate (4.5 mM sodium carbonate/1.4 mM sodium bicarbonate) was used.

**Fig. 3:**
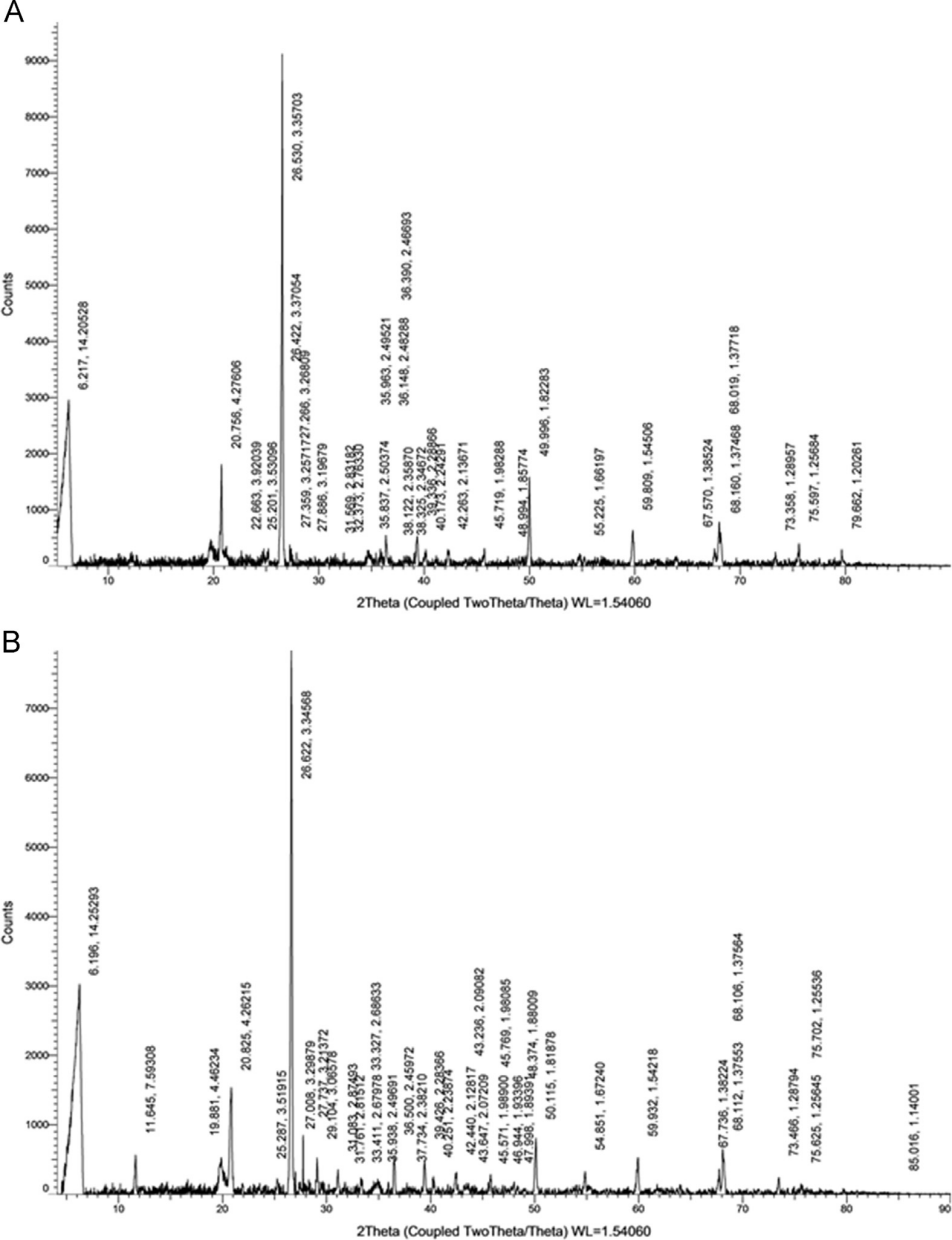
Powder XRD patterns of samples from the Nacogdoches Wastewater Treatment Plant (A), and Lufkin Wastewater Treatment Plant (B). The 2*θ* values and d-spacing values corresponding to each crystalline phases are also shown. The crystalline phases corresponding to each peak(s) are presented in Table 1, Table 2, respectively.

**Fig. 4:**
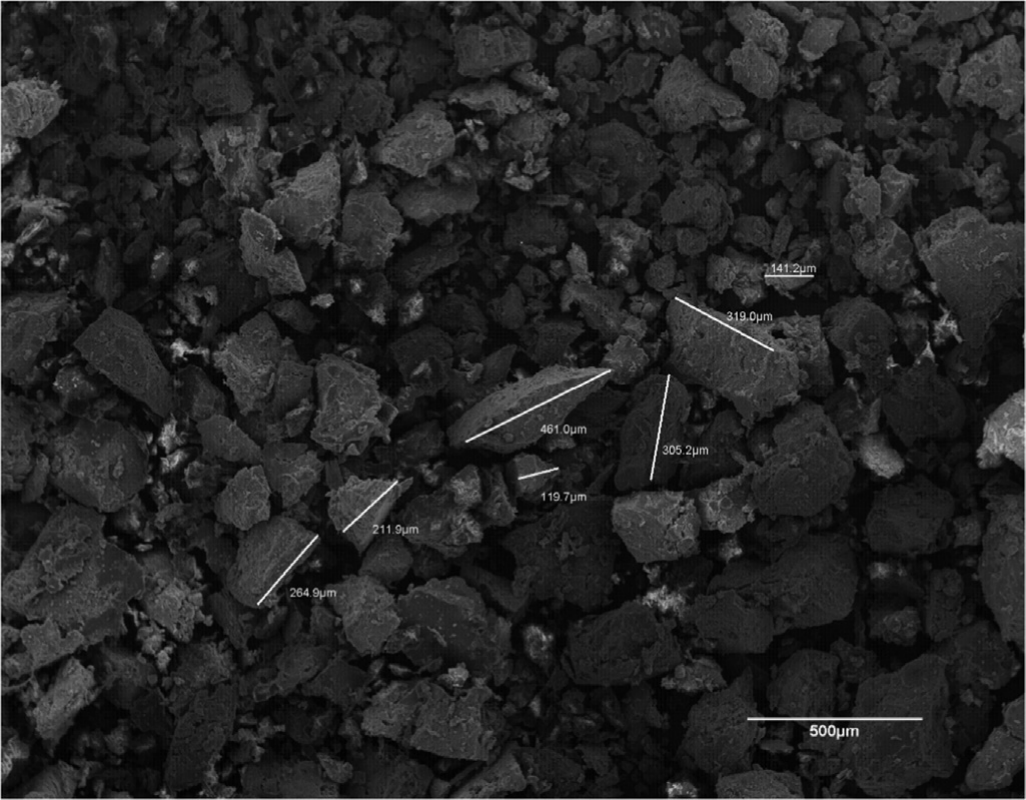
Representative SEM micrograph of LWWTP biosolid showing particle size diameters (magnification 40×, Voltage applied =15 kV).

**Fig. 5:**
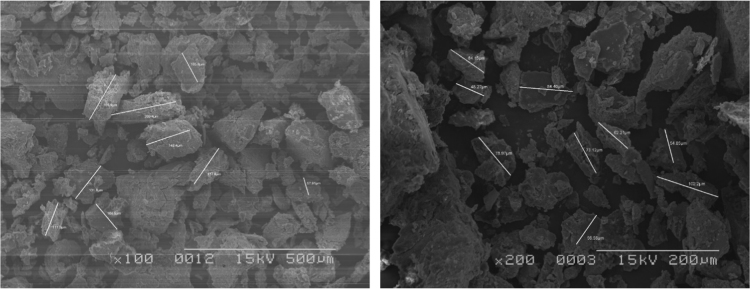
Representative SEM micrograph of the LWWTP biosolid with measurement of smaller particles; magnification 100×, and 200×.

**Fig. 6:**
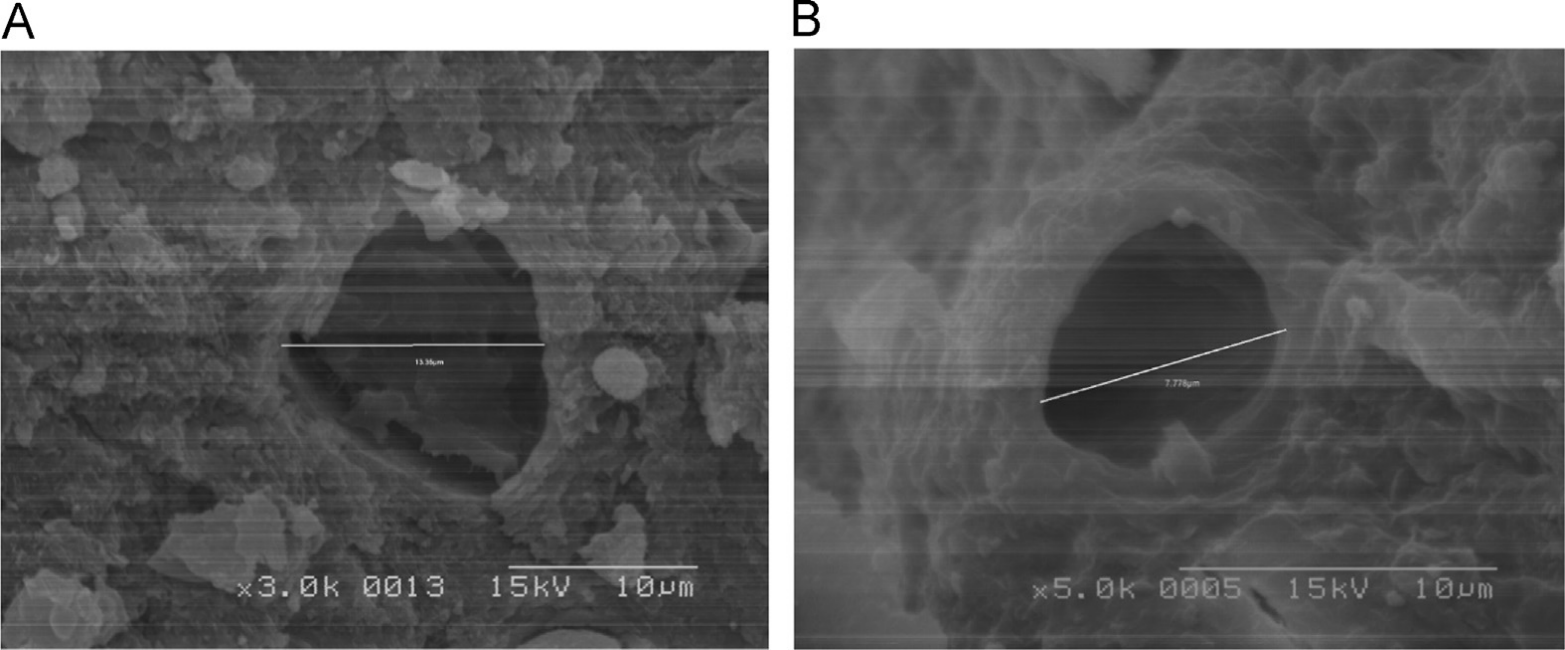
SEM micrograph of a pore from the NWWTP biosolid with measurement of its diameter; magnification 3 kV (a) and 5 kV (b).
